# Liver irradiation causes distal bystander effects in the rat brain and affects animal behaviour

**DOI:** 10.18632/oncotarget.6596

**Published:** 2015-12-15

**Authors:** Anna Kovalchuk, Richelle Mychasiuk, Arif Muhammad, Shakhawat Hossain, Slava Ilnytskyy, Abhijit Ghose, Charles Kirkby, Esmaeel Ghasroddashti, Olga Kovalchuk, Bryan Kolb

**Affiliations:** ^1^ Department of Neuroscience, University of Lethbridge, Lethbridge, AB, Canada; ^2^ Department of Biological Sciences, University of Lethbridge, Lethbridge, AB, Canada; ^3^ Jack Ady Cancer Center, Alberta Health Services, Lethbridge, AB, Canada; ^4^ Department of Physics and Astronomy and Department of Oncology, University of Calgary, Calgary, AB, Canada; ^5^ Alberta Epigenetics Network, Calgary, AB, Canada; ^6^ Canadian Institute for Advanced Research, Toronto, ON, Canada

**Keywords:** radiation therapy, brain, neuroanatomy, behaviour, gene expression

## Abstract

Radiation therapy can not only produce effects on targeted organs, but can also influence shielded bystander organs, such as the brain in targeted liver irradiation. The brain is sensitive to radiation exposure, and irradiation causes significant neuro-cognitive deficits, including deficits in attention, concentration, memory, and executive and visuospatial functions. The mechanisms of their occurrence are not understood, although they may be related to the bystander effects.

We analyzed the induction, mechanisms, and behavioural repercussions of bystander effects in the brain upon liver irradiation in a well-established rat model.

Here, we show for the first time that bystander effects occur in the prefrontal cortex and hippocampus regions upon liver irradiation, where they manifest as altered gene expression and somewhat increased levels of γH2AX. We also report that bystander effects in the brain are associated with neuroanatomical and behavioural changes, and are more pronounced in females than in males.

## INTRODUCTION

While ionising radiation (IR) is a key mainstream diagnostic and treatment modality, it is also a potent DNA-damaging agent capable of inducing a variety of diseases, including cancer. Today, radiation therapy (RT) is one of the main sources of IR exposure. Recent studies have proven that the brain is very sensitive to irradiation, and RT impacts a wide array of brain functions, causing cognitive decline, memory deficits, fatigue, and brain tumours in exposed individuals (reviewed in [1, 2]). The extent and severity of IR's effects on the brain depend upon its dosage [1]. While the effects of high doses of IR have been studied and are reasonably well understood, the effects and mechanisms of the brain's response to low doses of IR need to be analyzed in more detail [1, 3, 4].

Brain irradiation effects are specific to age, brain region, and sex [5-7]. Of the various brain regions, the prefrontal cortex and the hippocampus are particularly sensitive to IR [8-12]. The hippocampus is one of two active sites of neurogenesis in the mammalian brain [13]. The proliferation of neuronal precursors in the subgranular zone of the dentate gyrus generates cells that migrate further to the granule cell layer and differentiate into mature neuronal and glial phenotypes [14].

The prefrontal cortex (PFC) is a key regulatory region that collects input from all other cortical regions; it plans and directs an array of motor, cognitive, and social behaviours. The PFC receives inputs from the ventral tegmental area and connects with virtually all regions of the forebrain. It is susceptible to harmful factors like stress, which can lead to abnormal functioning [15].

Irradiation is a well-known cause of apoptosis, and it blocks neurogenesis in the dentate subgranular zone [16]. Such blockages persist for prolonged periods of time. The PFC is also very sensitive to different environmental stresses, including IR. IR exposure results in a loss of cells in the hippocampal CA1 subfield, reduces spine density and dendritic length in the dentate gyrus, and impacts gene and protein expression in the PFC [6, 12, 17, 18].

In addition, while it has been broadly accepted for several decades that the biological effects of radiation exposure are attributable to the direct damaging effects of irradiation in exposed tissues, this paradigm has been challenged by numerous experiments proving that even those cells that are not directly traversed by IR exhibit responses that are very typical of directly irradiated cells [19]. Such IR-induced ‘bystander’ effects have been seen in both naïve cells that were in contact with directly irradiated cells and naïve cells that received certain irradiation ‘distress’ signals from the directly exposed cells [20].

Cranial exposure causes a wide array of molecular bystander effects in animals' shielded spleens, livers, and gonads [21-23]. IR-induced bystander effects persist for a long time following irradiation. While cranial exposure has been shown to cause bystander effects in somatic organs, very little is known about the potential bystander effects caused by the irradiation of distal somatic organs on a shielded brain.

Here, we present the first evidence that bystander effects occur in the brain as a result of liver irradiation; these effects manifest as altered gene and protein expression and DNA damage. They are associated with neuroanatomical and behavioural changes, and are more pronounced in females than in males.

## RESULTS

### Liver irradiation model to study bystander effects in the brain

We studied bystander effects in the brain by exposing the liver of an experimental animal to IR while protecting the rest of the body with a medical-grade lead shield (Figure S1). We compared this result to the effects of head irradiation. Detailed dose analyses revealed that directly irradiated brains received doses of 24.5 centiGrays (cGy). When the liver was irradiated, the brain received a small scatter dose of 0.125 cGy [24]. Both doses belong to the low-dose radiation range.

### Persistence of DNA damage in exposed and bystander PFC tissues *in vivo*

**Figure 1:**
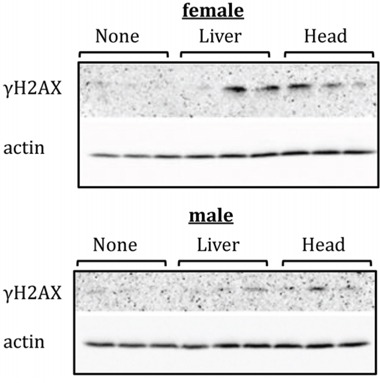
Levels of phosphorylated H2AX (γH2AX ) in PFC tissues of head- and liver-irradiated female and male animals Lysates from PFC tissues were immunoblotted using antibodies against γH2AX. Each sample represents an individual animal. All female samples were run on one gel under the same conditions; all male samples were run on another gel under the same conditions.

The induction of DNA damage constitutes a well-established bystander effect endpoint [25]. To analyse the levels of DNA damage, we assayed for the presence of H2AX phosphorylation in the hippocampus and PFC tissues of control, liver-exposed, and head-exposed animals. H2AX is a member of the H2A histone family that becomes phosphorylated at S139 (γH2AX) as one of the earliest cellular responses to double-strand breaks in DNA [26]. Two weeks after exposure, γH2AX was virtually undetectable by western blot in the PFCs of un-irradiated control rats, but was found, albeit in small amounts, in the PFCs of head-irradiated female and male rats (Figure 1). Furthermore, very small amounts of γH2AX were detected in the PFCs of female rats that had been subjected to liver irradiation, which could be indicative of increased levels of bystander DNA damage in these animals. No γH2AX was detected in the PFCs of liver-irradiated male rats. No changes in the γH2AX levels were seen in the hippocampi of head- or liver-exposed male and female rats (data not shown).

### Gene expression in the PFC and hippocampus tissues of control and exposed rats

Bystander effects often manifest as aberrant gene expression profiles. Therefore, we next analysed the global transcriptomes of the PFC and hippocampal tissues in male and female rats following liver irradiation using Illumina-based massively parallel sequencing. Transcriptome profiling revealed profound sex- and region-specific differences in gene expression. In males, liver irradiation affected one particular predicted gene in the hippocampus and PFC: ENSRNOG00000043197.

In females, twenty-two genes were differentially expressed in bystander PFC tissues following liver irradiation (Table 1). To further substantiate our results, we conducted western immunoblotting for the protein products of two differentially expressed genes: Tbx18 and Eaat2. Compared with the case of control rats, both genes were down-regulated in the bystander PFC tissues of the liver-exposed female rats. The levels of both the TBX18 and EAAT2 proteins were also lower in the PFC tissues of the liver-exposed female rats than in those of the control rats (Table 1, Figure S2).

**Table 1:** List of genes differentially expressed in the PFC of female rats upon liver irradiation. Levels of corresponding proteins were determined by western immunoblotting and were significant (p < 0.05)

Gene	Gene expression (Log2Fold)	Protein level (Fold)
Collagen alpha-2(I)	−1.21	
T-box transcription factor TBX18	−1.30	−1.09
Fibronectin Anastellin	−0.82	
Collagen alpha-1(I) chain	−1.00	
Excitatory amino acid transporter 2	−0.61	−1.24
Inactive carboxypeptidase-like protein X2	−1.13	
Insulin receptor substrate 2	−0.75	
ENSRNOG00000024374|Uncharacterized protein	−0.55	
Adenylate cyclase type 1	−0.65	
Putative RNA-binding protein 3	0.52	
Biregional cell adhesion molecule-related/down-regulated by oncogenes (Cdon) binding protein	−0.57	
Collagen alpha-1(III) chain	−1.20	
Cadherin-1E-Cad/CTF1E-Cad/CTF2E-Cad/CTF3	−1.55	
Retinal dehydrogenase 2	−0.81	
Insulin-like growth factor-binding protein 2	−0.77	
Pecanex-like protein 1	−0.40	
ENSRNOG00000001249|Uncharacterized protein	−1.03	
SLIT-ROBO Rho GTPase-activating protein 3	−0.40	
Fibulin-1	−0.72	
Tubulin polymerization-promoting protein	−0.47	
Collectin-12	−0.91	
ENSRNOG00000019462|Uncharacterized protein	−0.39	

### Neuroanatomical changes induced by head and liver irradiation

**Figure 2:**
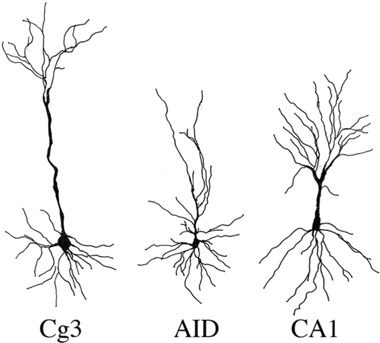
Representative samples of camera lucida drawings of pyramidal neurons used for spine density and dendritic analysis in medial prefrontal cortex (Cg3), orbital frontal cortex (AID), and hippocampus (CA1) of male and female rats exposed head or liver irradiation

**Figure 3:**
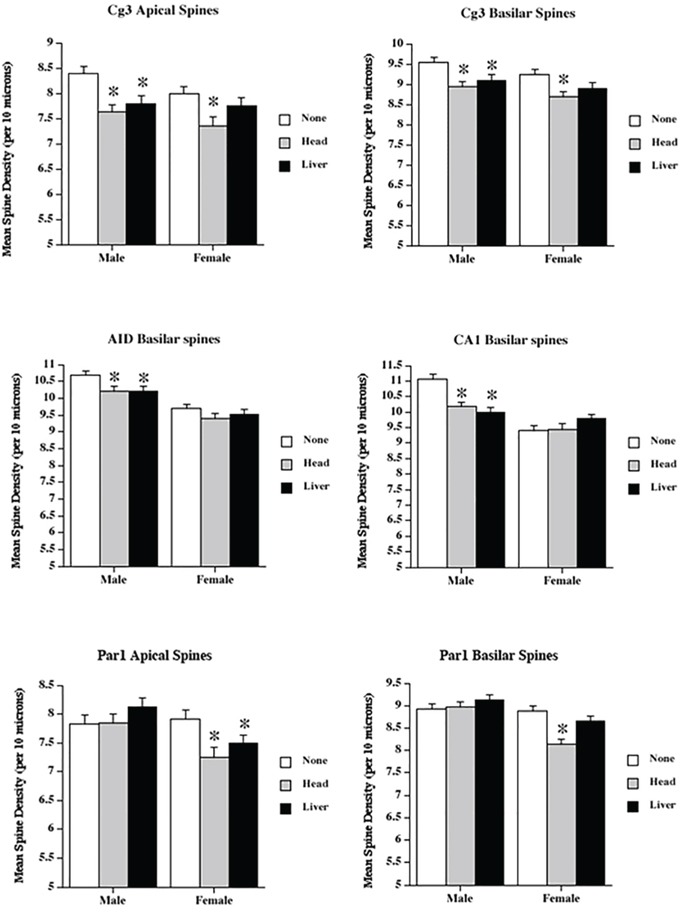
Low dose radiation exposure affects spine density The density of dendritic spines (spines/10μM) in medial prefrontal cortex (Cg3), orbital frontal cortex (AID), parietal cortex (Par1), and hippocampus (CA1) of male and female rats upon head or liver irradiation. *Significantly different from the control unexposed animals.

**Figure 4:**
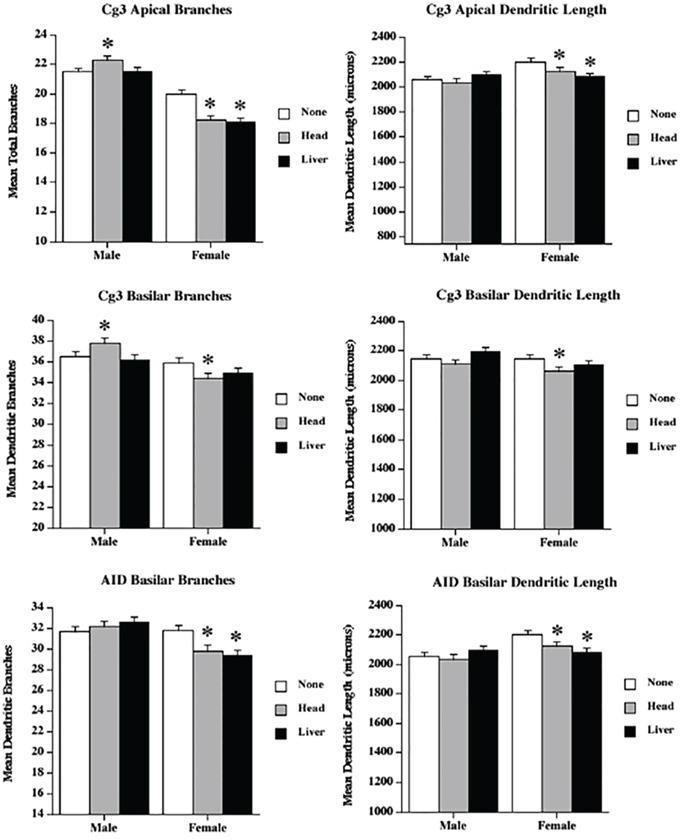
Low dose radiation exposure causes changes in dendritic branching and length Apical and basilar branching and dendritic length in medial prefrontal cortex (Cg3) and orbital frontal cortex (AID) of male and female rats upon head or liver irradiation. *Significantly different from the control unexposed animals *p* < .05 or better.

Because we had witnessed slightly increased levels of γH2AX and altered gene expression, we next analysed the neuroanatomical characteristics of the brains of head- and liver-irradiated animals. Our overall finding was that radiation applied to the liver or the head produced extensive changes in dendritic organisation in all regions measured, with the effects being most extensive in the PFC. The effects were greater in the head irradiation group than in the liver irradiation group. Many of the effects were sexually dimorphic, as the females tended to be more affected than the males. In most measures, the males (including the control males) had higher values than the females. We considered each region in turn (see Figure 2 for examples of the neurons from Cg3, AID, and CA1; Figure 3 for spine data; and Figure 4 for dendrite data). Note that only the basilar fields were drawn for AID and CA1, whereas both the apical and basilar fields were drawn for Cg3 and Par1.

### Medial prefrontal cortex (Cg3)

The greatest effects of irradiation were seen in Cg3 in both sexes. Two-way analysis of variance (ANOVA) was run for all analyses, with ‘Treatment’ and ‘Sex’ as factors (F).

#### Apical field spine density

Spine density was reduced in both irradiation groups. ANOVA revealed significant effects for treatment (F[2,68] = 12.5, *p* < .001), and sex (F[1,68] = 3.83, *p* = .05), but not their interaction (F[2,68] = 0.66, *p* = .52). The sex difference reflected greater spine density in males than in females.

#### Apical field branching

Apical branching was reduced in females, but increased in the male head irradiation group. ANOVA revealed significant effects for treatment (F[2,68] = 6.3, *p* = .003), sex (F[1,68] = 187.9, *p* < .001), and their interaction (F[2,68] = 12.25, *p* < .001). The sex difference reflected greater branching in males than in females, and the interaction reflected opposite effects of head irradiation in the two sexes.

#### Apical field dendritic length

Irradiation reduced dendritic length following both types of irradiation. ANOVA revealed significant effects for treatment (F[2,68] = 8.9, *p* < .001), sex (F[1,68] = 56.3, *p* < .001), and their interaction (F[2,68] = 3.6, *p* = .03). Males had longer dendrites than females, and the effect of radiation was larger in females than in males.

#### Basilar field spine density

Spine density was reduced in both irradiation groups. ANOVA revealed significant effects for treatment (F[2,68] = 11.3, *p* < .001) and sex (F[1,68] = 5.3, *p* = .02), but not for their interaction (F[2,68] = 1.4, *p* = .88). The sex difference again reflected greater spine density in males than in females.

#### Basilar field branching

The results for the basilar field branching were similar to those for the apical branching, with sexually dimorphic irradiation effects. ANOVA revealed no significant effect for treatment (F[2,68] = 11.15, *p* = .32), but showed significant effects for sex (F[1,68] = 22.12, *p* < .001) and the interaction (F[2,68] = 5.0, *p* = .009). The sex difference resulted from the existence of more complex neurons in the males, and the interaction reflected an increase in branching in the male head irradiation group (compared to a decrease in both female irradiation groups).

#### Basilar field dendritic length

The effects on basilar length were complex: the head irradiation reduced length, but the liver irradiation did not. ANOVA revealed significant effects for treatment (F[2,68] = 3.02, *p* = .05) and sex (F[1,68] = 3.93, *p* = .05), but not for their interaction (F[2,68] = 0.12, *p* = .25).

### Orbital prefrontal cortex (AID)

#### Basilar field spine density

Spine density was reduced overall from irradiation, but was higher in males. ANOVA revealed significant effects for treatment (F[2,68] = 4.7, *p* < .013) and sex (F[1,68] = 51.8, *p* < 001), but not their interaction (F[2,68] = 0.6, *p* = .56).

#### Basilar field branching

Although ANOVA revealed no effects for treatment (F[2,68] = 1.39, *p* = .25), there were significant effects for sex (F[1,68] = 20.9, *p* < 001) and the interaction (F[2,68] = 6.68, *p* = .002). The interaction resulted from a significant drop in branching in the females, but not in the males. Once again, males had more complex cells than females.

#### Basilar field dendritic length

As in the branching results, ANOVA revealed no effects of treatment (F[2,68] = 1.8, *p* = .17); there were, however, significant effects for sex (F[1,68] = 9.8, *p* = 003) and the interaction (F[2,68] = 4.4, *p* = .02). Moreover, as in the branching, the interaction resulted from a significant drop in length in the females, but not in the males.

### Hippocampus (CA1)

The effects of irradiation in the hippocampus were surprisingly small, relative to those found in the prefrontal regions, and were seen only in males.

#### Basilar field spine density

Irradiation significantly reduced spine density in males, but not in females. In addition, as in other regions, males had higher spine densities than females. ANOVA revealed significant effects for treatment (F[2,68] = 4.1, *p* = .02), sex (F[1,68] = 2.7, *p* = .07), and their interaction (F[2,68] = 9.3, *p* < .001).

#### Basilar field branching

There were no significant effects of irradiation on branching. ANOVA found no effects for treatment (F[2,68] = 0.58, *p* = .56), sex (F[1,68] = 2.2, *p* = .14), or their interaction (F[2,68] = 1.4, *p* = .88).

#### Basilar field dendritic length

There was a significant effect of head irradiation, but not liver irradiation, on dendritic length. ANOVA found an effect for treatment (F[2,68] = 3.02, *p* = .05), but not for sex (F[1,68] = 0.00, *p* = 1.00) nor the interaction (F[2,68] = .15, *p* = .6). Although there was a trend of shorter dendrites in both irradiation groups, it was only significant in the head groups.

### Parietal cortex (Par1)

#### Apical field spine density

There was a significant effect (reduced spine density) of irradiation in females, but not in males. ANOVA showed no significant effect for treatment (F[2,68] = 2.24, *p* = .12), but did show effects for sex (F[1,68] = 8.7, *p* = .004) and the interaction (F[2,68] = 3.68, *p* = .03). The interaction reflected the decrease in females, but not in males.

#### Apical field branching

There was no effect of irradiation in either sex, although males had more branching than females. ANOVA found no significant effects for treatment (F[2,68] = 1.45, *p* = .24) or the interaction (F[2,68] = 2.05, *p* = .137), but did show a significant effect for sex (F[1,68] = 4.5, *p* = .037).

#### Apical field dendritic length

There were no significant effects of either sex or irradiation on apical dendritic length. ANOVA showed no significant effects for treatment (F[2,68] = 0.96, *p* = .39), sex (F[1,68] = .21, *p* = .65), or the interaction (F[2,68] = 0.75, *p* = .45).

#### Basilar field spine density

As with the apical spines, there was a significant reduction from irradiation in females, but not in males. ANOVA showed significant effects for treatment (F[2,68] = 6.29, *p* = .003), sex (F[1,68] = 25.0, *p* < .001), and their interaction (F[2,68] = 6.75, *p* = .002). The interaction reflected the decrease in females, but not in males.

#### Basilar field branching

There was an unexpected effect of an increase in branching in the head irradiation groups. ANOVA (Treatment x Sex) found an effect for treatment (F[2,68] = 3.01, *p* = .05), but not for sex (F[1,68] = 0.19, *p* = .66) or the interaction (F[2,68] = .342, *p* = .71).

#### Basilar field dendritic length

There were no significant effects of either sex or irradiation on basilar dendritic length. ANOVA showed no significant effects for treatment (F[2,68] = 1.02, *p* = .37), sex (F[1,68] = 1.36, *p* = .25), or their interaction (F[2,68] = 0.37, *p* = .69).

### Behavioural changes induced by head and liver irradiation

**Figure 5:**
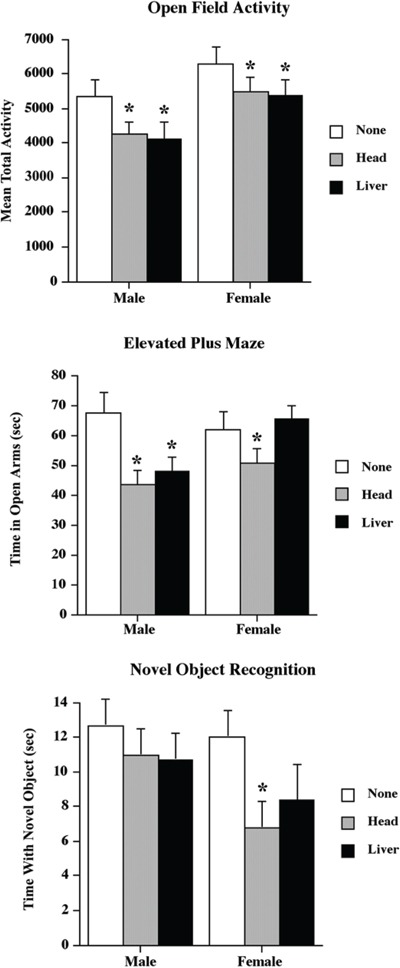
Low dose head and liver irradiation exposure affect animal behavior Graphical representation of the behavioral data for the Open Field Activity, elevated plus maze and Novel Object Recognition tests. *Significantly different from the control unexposed animals *p* < .05 or better.

Overall, both head and liver radiation affected animal behaviour in both the activity test and the elevated plus maze (EPM), but not in the novel object recognition test (Figure 5), which is a test of memory. We considered each separately.

#### Activity

Both head and liver irradiation significantly reduced activity, with the reduction being about 20 percent in males and 15 percent in females (see Figure 5). A two-way ANOVA found a significant effect for treatment (F[2,25] = 3.90, *p* = .03) and sex (F[1,40] = 10.78, *p* < .01), but not for their interaction (F[2,35] = .08, *p* = .92).

#### Elevated plus maze

Head irradiation significantly reduced the time spent by both male and female animals in the open arms of the maze (described further in the Behavioural Analysis section below), but liver irradiation only reduced activity in males (Figure 5). A two-way ANOVA found a significant effect for treatment (F[2,35] = 6.07, *p* < .01), but not for sex (F[1,40] = 2.35, *p* = .13). It also found a nonsignificant trend toward an interaction (F[2,35] = 2.69, *p* = .08). The interaction trend reflected the absence of a treatment effect in the female liver radiation group.

#### Novel object recognition

There were no significant effects of radiation on novel object recognition (Figure 5). A two-way ANOVA found no significant effects for treatment (F[2,25] = 1.62, *p* = .21), sex (F[1,40] = 1.95, *p* < .17), or their interaction (F[2,35] = 2.37, *p* = .29). However, inspection of Figure 5 suggests a trend toward a reduced amount of time spent with the novel object in the female radiation groups. Given this observation, we performed a posthoc t-test comparing the female head irradiation and non-irradiation groups and found a significant difference (p < .05).

## DISCUSSION

This study is the first to conduct a large-scale analysis of the molecular, neuroanatomical, and behavioural consequences of direct and bystander low-dose irradiation on the rodent brain. The key findings of this study are that: 1) direct head exposure to 24.5 cGy causes persistent albeit small increase in DNA damage as measured by the slightly elevated levels of γH2AX and affects gene expression in the PFCs of exposed animals; 2) bystander effects of liver exposure to a dose as low as 0.125 cGy in the shielded bystander brain manifest as a slight increase in the levels of γH2AX and as altered gene and protein expression; 3) both head and liver irradiation reduce dendritic space (and, thus, synapse number) in measures of spine density, dendritic complexity, and dendritic length; 4) the neuroanatomical effects are brain-region-specific, and are more pronounced in females; and 5) both head and liver irradiation alter behaviour.

The direct low-dose IR and the bystander-induced DNA damage in the brain represent an interesting observation. We analysed the DNA damage by studying the levels of phosphorylated histone H2AX (γH2AX). γH2AX is a well-accepted marker of DNA strand breaks [26], and bystander cells were reported to exhibit an accumulation of γH2AX [27].

Our study is the first to show the presence of γH2AX in the shielded bystander brains. Two weeks after exposure, we found the presence of γH2AX in the PFCs of female rats subjected to head and liver irradiation, which suggests increased albeit small levels of direct and bystander DNA damage in these animals. This may be due to inefficient DNA repair, or to the fact that small amounts of damage may be simply overlooked by DNA repair systems. In the future, it will be important to analyse and confirm the precise nature of the DNA damage caused by direct and bystander irradiation in the PFC.

Although low-dose radiation effects, as well as bystander effects, have been shown to cause changes in gene expression in affected cells [28], nothing was previously known about the effects of liver irradiation on gene expression in distal bystander brain tissues. In this study, we analysed the global transcriptomes of PFC and hippocampal tissues in male and female rats following liver irradiation and uncovered interesting sex- and brain-region-specific changes in gene expression. In males, liver irradiation affected one predicted gene in the hippocampus and PFC: ENSRNOG00000043197. This locus is a predicted target of several transcription factors, such as CdxA, GATA-1, SRY, HFH-2, and p300. Interestingly, SRY is the sex determination factor that is expressed only in males, which may explain why this gene is up-regulated in a sex-specific manner. Further analysis is needed to explore this gene, its function, and its regulation in detail.

For the first time, we noted significant and sex-specific alterations in gene expression profiles, in which twenty-two genes were affected in female PFCs (Table 1). Differentially expressed genes included those involved in the function of the blood-brain barrier, neuroinflammation, and apoptosis. Amongst these there was the Eaat2 gene. Its altered expression were also confirmed to exist on the protein level. EAAT2 belongs to excitatory amino-acid transporters, which are also known as glutamate transporters, a family of neurotransmitter transporters that affect brain function and development. EAAT2 is also one of the major glutamate transporters expressed in astroglial cells that governs approximately 90 percent of total glutamate uptake. Loss of EAAT2 function has been associated with the development of neurodegenerative diseases, such as Alzheimer's and Huntington's diseases, amyotrophic lateral sclerosis, and malignant glioma [29, 30]. The down-regulation of EAAT2 therefore may have negative consequences on PFC function. The roles of EAAT2 in direct and bystander radiation responses need to be further explored.

Our study found that liver irradiation also negatively affects the expression of collagens in the bystander PFCs of female animals. Collagens protect against neuronal apoptosis and are engaged in the blood-brain barrier function [20]. Previous studies have reported that exposure to 10 or 40 Gy of γ-rays leads to reduction in collagen levels and dysfunction of the blood-brain barrier [31]. Additionally, we noted decreased levels of Slit-Robo Rho GTPase-activating protein 3 and Pecanex-like protein 1. Pecanex exerts a neurogenic role in Drosophila [32]. The Slit-Robo pathway has been shown to play a role in axonal regeneration after nerve injury [33]. Moreover, disruption of some of the Slit-Robo Rho GTPase-activating proteins has been linked to the development of infantile epileptic encephalopathy [34]. The roles of these proteins in low-dose radiation and bystander effects also need to be further examined.

Most importantly, our study showed extensive bystander effects in brain morphology, which manifested as decreased spine density, dendritic length, and dendritic complexity in the two PFC regions measured, the parietal cortex, and the hippocampus. These effects were generally more profound in females, and the largest effects were found in the PFC regions, with surprisingly small effects seen in the hippocampus. While previous studies have shown conclusively that irradiation interferes with neurogenesis, leading to cognitive impairment [12, 18], still fairly little is known about the effects of irradiation on mature neurons. We chose to examine dendritic measures in several brain regions that did not have adult neurogenesis. The sizes of dendritic fields and the densities of the spines provide indirect measures of synaptic organisation and number. Reductions in these measures are correlated with several brain disorders, including dementia, Down syndrome, and fragile-X syndrome, and changes (e.g., increases or decreases in the synaptic space) have been associated with learning [35]. Parihar and Limoli [18] showed in a study on male mice that higher levels of cranial irradiation than those used in the current study reduced spine density and dendritic complexity in the dentate gyrus of the hippocampus. Their observed effects were much larger than those observed in this study, which used much lower irradiation doses and looked at CA1. Chakraborti et al. [36] also studied spine densities in male mice, and found decreased spine densities in both the dentate gyrus and CA1 of the hippocampus. Most recent study by Pahirar et al. established that low, space-relevant doses of charged particles reduced dendritic complexity and spine density in PFC of male mice [12].

Thus, the novel neuroanatomical findings here are that: 1) both direct head and indirect (bystander-to-liver) irradiation in low doses reduces synaptic space in both the hippocampus and the neocortex, and 2) these changes are sexually dimorphic and area-specific. Indeed, the effects in both prefrontal regions measured were far more extensive than those seen in the hippocampus.

The current study also showed that neuroanatomical and brain molecular changes were associated with behavioural changes, as well. The decrease in activity associated with both head and bystander irradiation was unexpected, given that lesions to the PFC or the hippocampus tend to increase activity [37]. The irradiated animals did not have lesions; instead, they experienced a reorganisation of circuits, which presumably accounts for the difference. The irradiation also increased anxiety, a result commonly associated with stressful experiences. Curiously, the bystander effect on anxiety was only observed in males, possibly reflecting the male-specific changes in spine density in both AID and CA1. Finally, the novel object recognition test, which is a test of memory, showed head irradiation to have an effect on females, but not on males. This sexually dimorphic effect may be associated with the decrease in dendritic branching and length found in female, but not male, Cg3 neurons. Lesions of the medial PFC are associated with deficits in temporal order memory, such as the memory used for current tasks [38].

Bystander effects in the brain were previously shown in two studies by Mancuso and colleagues [39, 40], who analysed changes in the cerebellums of two mutant mouse strains following irradiation of the animal bodies. In our study, we also observed bystander effects in the cerebellum, which manifested as altered levels of protein expression (data not shown). The changes in the cerebellum, however, were less pronounced than those in the PFC and the hippocampus. Moreover, our study engaged in a detailed analysis of the scatter dose received by the brain during liver exposure. The dose (0.125 cGy) was very low, nevertheless it could cause some of the observed effects. Low doses were previously shown to exert strong mutagenic potential on exposed cells and tissues. Aside from the small scatter dose, a blood-borne bystander signal may also exist. Such a signal may originate in exposed blood cells and spread via the blood. Indeed, numerous blood cells are located in the exposure field during the exposure of the liver (which received 30 cGy). Blood cells are sensitive to irradiation and can undergo apoptosis or necrosis. They then release a variety of soluble factors that are small enough to cross the blood-brain barrier and damage it. The molecular identity of the bystander factors needs to be defined, and the roles played by these factors, and by small scatter doses in bystander effects, should be further investigated.

## MATERIALS AND METHODS

### Animal model and tissue sampling

Forty four male and forty four female three-month-old Long Evans rats (Charles River) were used in this study. Sixteen male and sixteen female animals were used for molecular profiling, and twenty eight male and twenty eight female animals were used for neuroanatomical analysis and behavioural testing.

The animals were housed in a pathogen-free controlled facility with a 12 h light/dark cycle and given food and water ad libitum. The animals were randomly allocated to the following groups: head-exposed, liver-exposed and sham-treated control, as described before [24]. For irradiation, the animals were anaesthetised through intra-peritoneal injections of ketamine/xylazine (50/5 mg/kg). The anaesthesia was well tolerated, and no side effects were observed. Head- and liver-exposed animals received X-ray irradiation delivered to the surface of the respective area of their body; a medical-grade lead shield protected the rest of the body. Specifically, for each target organ, a lead apron (0.05 cm Pb-equivalent) was used for shielding. A 1.7 cm by 3.5 cm oval was cut into the apron in order to define a primary field, and the apron was then placed on the rats. The doses delivered to the respective organs were determined as follows: for head exposure, a dose to the brain constituted 24.5 cGy. In the liver-exposed/brain bystander scenario, the centre-to-centre distances between organs were roughly 6.0 cm brain-to-liver. During liver exposure, the brain was shielded, but it still received a dose of 0.125 cGy due to radiation scattering [24]. The handling and care of the animals were conducted in accordance with the recommendations of the Canadian Council for Animal Care and Use. The University of Lethbridge Animal Welfare Committee approved all procedures.

### Molecular profiling

For molecular analysis, the animals were euthanized 14 days after irradiation. Upon sacrifice, the brain areas (hippocampus and prefrontal cortex) were sampled and snap-frozen.

#### Gene expression analysis

The hippocampus and prefrontal cortex tissues of three animals per group were used for the analysis of the gene expression profiles. RNA was extracted using TRIzol® Reagent (Invitrogen, Carlsbad, CA); purified using an RNAesy kit (Qiagen), according to the manufacturer's instructions; and quantified using Nanodrop2000c (ThermoScientific). Next, RNA concentration and integrity were determined using 2100 BioAnalyzer (Agilent). Sequencing libraries were prepared using Illumina's TruSeq RNA library preparation kits. Gene expressions were determined using the Illumina deep sequencing platform at the University of Lethbridge CFI-SAGES Facility.

Statistical comparisons between the control and exposed groups within each tissue type were performed using the DESeq Bioconductor package (version 1.8.3) and the baySeq Bioconductor package (version 1.10.0). The clustering of the samples was assessed using multidimensional scaling (MDS) plots built using the plotMDS function of the edgeR Bioconductor package. MA plots showing the relationship between the average level of expression and the log2 fold change were built for each of the comparisons. Features with false discovery rates (FDR) < 0.1 (10% false positive rate) were considered differentially expressed between conditions.

#### Western immunoblotting

All female samples were run on one gel under the same conditions; all male samples were run on another gel under the same conditions. Western immunoblotting was conducted as described previously [6].

Membranes were stained overnight using primary antibodies against γH2AX (1:500, Cell Signaling, Danvers, MA), TBX18 and EAAT2 (1:1000, Abcam, Toronto, ON), and actin (1:2000, Abcam, Toronto, ON). Primary antibody binding was detected using horseradish peroxidase-conjugated secondary antibodies and the Enhanced Chemiluminescence Plus System (Amersham Biosciences, Baie d'Urfe, Quebec). Chemiluminescence was detected using a FluorChem HD2 camera with FluorChem software (Cell Biosciences); gel images were saved and processed using Adobe Professional under the same conditions. Bands corresponding to antibody binding in all samples were carefully cropped; no images were spliced. The membranes were stained with Coomassie blue (BioRad, Hercules, CA) to confirm equal protein loading. Signals were quantified using the NIH Image J64 software and normalized relative to actin or Coomassie staining.

### Histological processing and neuroanatomical analysis

For neuroanatomical analysis rats were given an overdose of sodium pentobarbital solution i.p. and perfused with 0.9% saline solution intracardially 14 days after exposure. Their brains were removed from their skulls, weighed and preserved in Golgi-Cox solution for 14 days, followed by transfer to 30% sucrose solution. The brains were sliced at a thickness of 200 μm on a vibratome and fixed on gelatinized slides. The slides mounted with brain sections were processed for Golgi-Cox staining, following the protocol described [41].

Pyramidal cells were drawn from layer 3 of Cg3 and AID (medial and orbital prefrontal regions, respectively) and from the CA1 region of the hippocampus, according to Zilles' cortical atlas [42]. Individual neurons were traced using a camera lucida mounted on a microscope. For dendritic branching and length, a total of 10 cells (5/hemisphere) were traced at 250X for each brain region. The averages of the cells from each hemisphere comprised the data points used for statistical analysis. Spine density was measured at 1000x and calculated by counting the number of spines on a length of distal dendrite that was least 50 microns in length. The exact length of the dendrite segment was calculated, and the density was expressed per 10 μm. Five segments were drawn per hemisphere from different neurons, and a mean value was calculated to use as the unit of measurement [43].

Branch order, which is an estimate of dendritic complexity, was used to measure the number of dendritic bifurcations. Dendritic length was calculated using a Sholl analysis, which is an estimation of dendritic length that counts the number of dendritic branches that intersect concentric circles spaced 25 um apart. Length is estimated by multiplying the number of dendritic intersections by 25.

### Behavioural analysis

Testing occurred two weeks after exposure.

#### Activity

The activity levels of the rats were measured two weeks after irradiation [44]. The rats were placed into an Accusan activity-monitoring box. The system consisted of an electronically fitted Plexiglas box measuring 41 × 41 × 30.5 cm that recorded the movements of each rat. The rats were left in the boxes for 10 min., and their exploratory behaviours were recorded in five 2-min intervals. The data were recorded using the VersaMax TM computer software. The five intervals were summed, and the results reported as the average of the total activity/distance travelled.

#### Elevated plus maze

Approximately three weeks after irradiation, the rats were tested in the elevated plus maze (EPM) [44]. The EPM was constructed from black Plexiglas, with a base measuring 94 cm high, two open arms measuring 10 cm wide and 40 cm long and two closed arms measuring 10 cm wide, 40 cm long, and 40 cm high. The maze was located in an empty room, and filming occurred with the lights on. The camera for filming was placed at the end of one of the open arms in a slightly elevated position. Filming occurred for five minutes. Rats were placed with their front paws in the center of the square maze facing a closed arm. Their performances were scored by a researcher blinded to experimental conditions, who measured the time spent in the open arms and the time spent in the closed arms. Reduced time spent in the open arms was taken as a measure of anxiety.

Testing was performed as described [45] and occurred two weeks after exposure. In brief, an NOR for temporal order memory was run in three separate trials, each starting one hour apart, on filming day. The rats were placed in a 48 cm × 48 cm × 52 cm white plastic container for five minutes three days prior to filming to habituate them to the testing conditions. On filming day, the initial trial involved placing two identical objects in the base of the tub. The animals were then left to explore the objects for four minutes. The second trial began one hour later and involved placing different identical objects in the tub with the animals for four minutes. The third trial consisted of placing the rats in the plastic container with one object from the first trial and one object from the second trial for four minutes. The time spent with each of the objects was calculated in the third trial. A rat was considered to be in contact with an object if its nose was within 2 cm of the object.

### Statistical analysis

All statistical analyses were carried out using SPSS 16.0 [45]. Each rat was used as a unit of analysis. Two-way ANOVAs using treatment (control, head-exposure, liver exposure) and sex (M/F) as factors were run to compare the behavioral outcomes in control and exposed rats.

## SUPPLEMENTARY FIGURES


