# Supplementary material for: Liver irradiation causes distal bystander effects in the rat brain and affects animal behaviour
Source: Oncotarget. 2015 Dec 15;7(4):4385–98. doi: 10.18632/oncotarget.6596 (PMC4826213; doi:10.18632/oncotarget.6596)
Supplement: Supplementary file 1 [file oncotarget-07-4385-s001.pdf]

SUPPLEMENTARY FIGURES

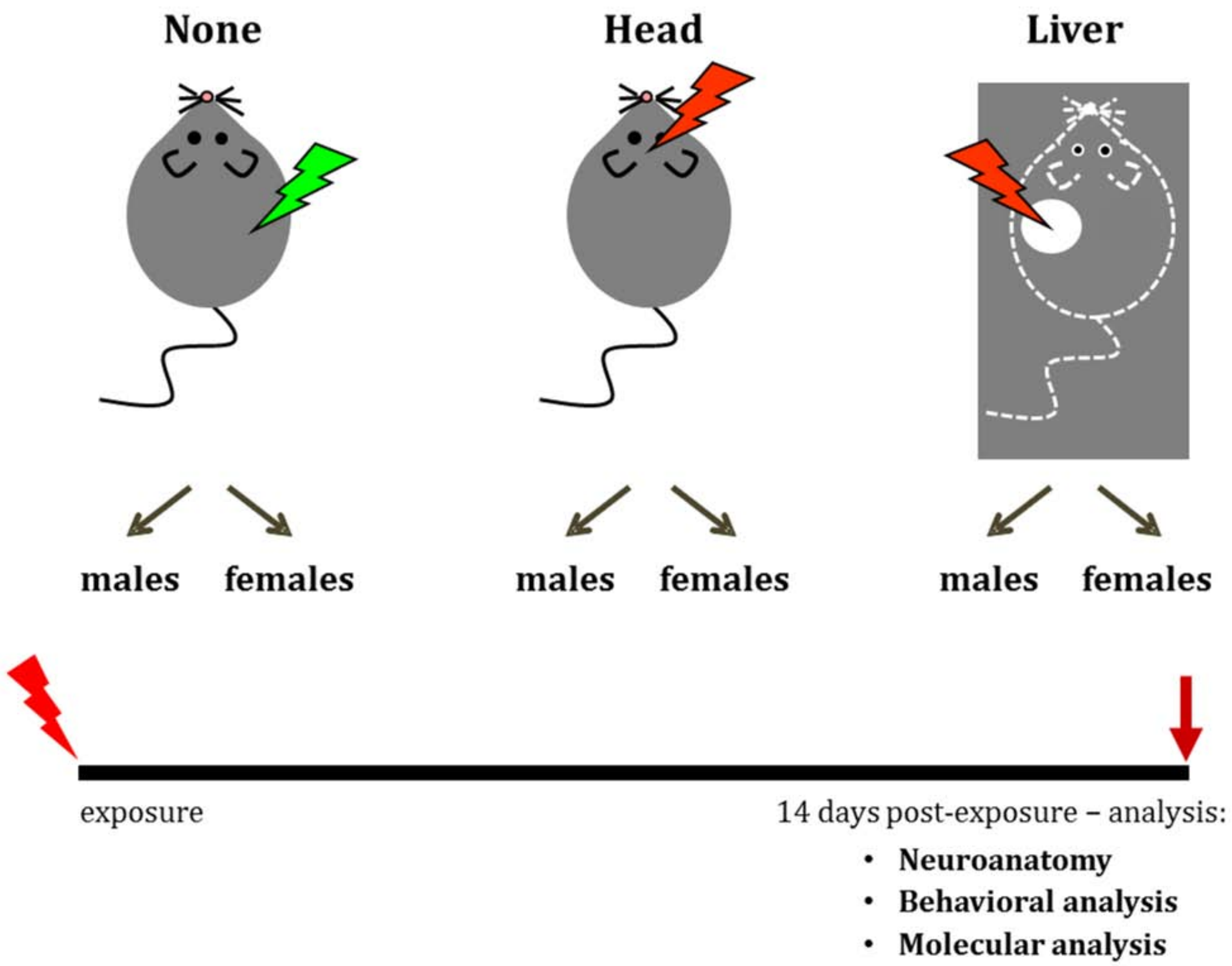

Supplementary Figure S1: Liver irradiation model to study bystander effects in the brain.

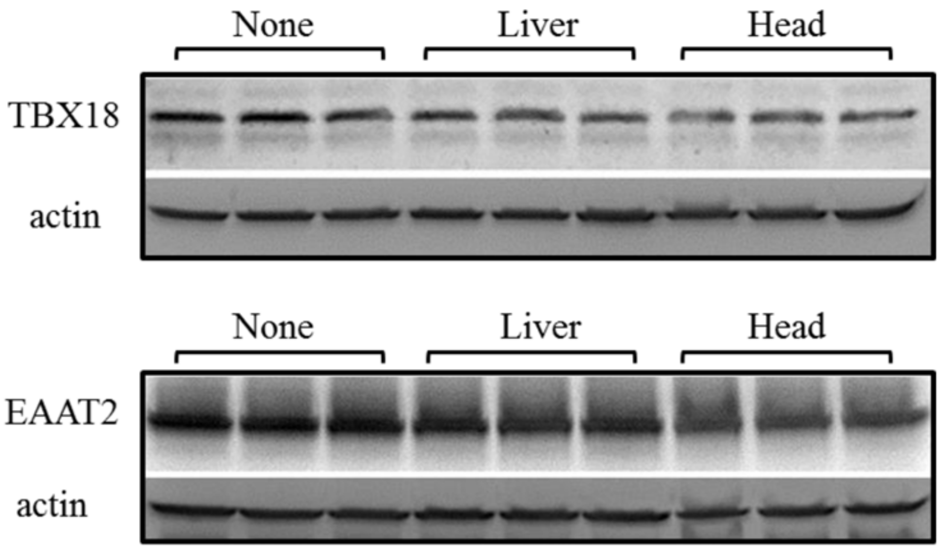

Supplementary Figure S2: Levels of TBX18 and EAA2 in PFC tissues of head- and liver-irradiated female animals. Lysates from PFC tissues were immunoblotted using antibodies against TBX18 and EAA2. Each sample represents an individual animal. All female samples were run on one gel under the same conditions; all male samples were run on another gel under the same conditions.

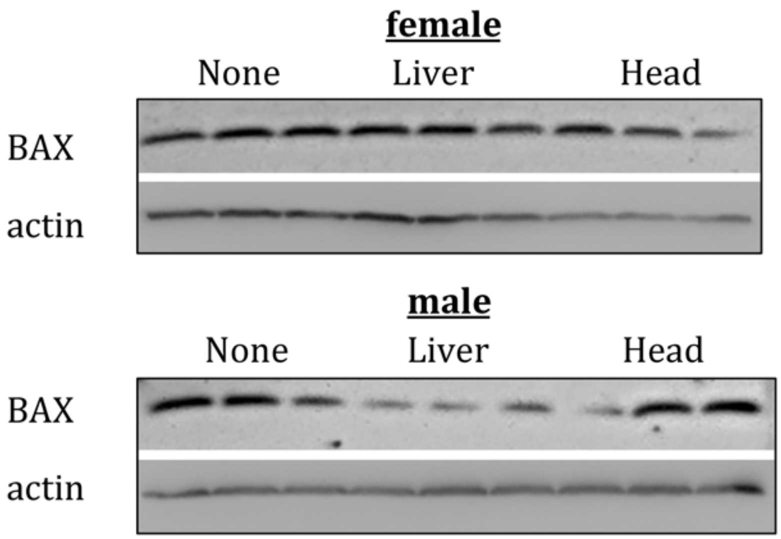

Supplementary Figure S3: Levels of BAX 2 in PFC tissues of head- and liver-irradiated female animals. Lysates from PFC tissues were immunoblotted using antibodies against BAX. Each sample represents an individual animal. All female samples were run on one gel under the same conditions; all male samples were run on another gel under the same conditions.
